# Supplementary material for: Protocol for a systematic review and meta-analysis of tobacco-cessation interventions delivered perioperatively
Source: BMJ Open. 2023 Sep 15;13(9):e067722. doi: 10.1136/bmjopen-2022-067722 (PMC10510911; doi:10.1136/bmjopen-2022-067722)
Supplement: Supplementary data [file bmjopen-2022-067722supp001.pdf]

Appendix 1: Search strategy

The search has been developed with the help of a librarian. A draft of the search is as follows:

Ovid MEDLINE(R) ALL <1946 to July 2022>

1 smoke*.ti,ab. 147122

2 "Tobacco Use Disorder"/ 12064

3 exp Smoking/ 158059

4 Tobacco/ or exp Tobacco Products/ 42941

5 tobacco.ti,ab. 110972

6 exp Smoking Devices/ 15555

7 (cigarette* or "e-cigarette*" or "electronic cigarette*" or vape* or vap?ing or cigar?).ti,ab. 80944

8 exp pipe smoking/ or exp tobacco smoking/ or vaping/ 153721

9 1 or 2 or 3 or 4 or 5 or 6 or 7 or 8 319299

10 (reduc* or quit* or stop* or abstin* or abstain* or cessat* or prevent* or consum* or program or intervention or "cut* down" or "cut-down" or "cut back" or "cut-back" or "cold turkey" or control* or schedul*).ti,ab. 9267831

11 9 and 10 167244

12 Smoking Cessation/ 31670

13 "Tobacco Use Cessation"/ 1386

14 exp "Tobacco Use Cessation Devices"/ 2355

15 ((Nicotine or tobacco) adj5 (replacement or cessation or patch* or "chewing gum" or inhalator* or tablet* or "oral strip*" or lozenge* or spray*)).ti,ab. 10237

16 Varenicline/ 1478

17 (Varenicline or champix).ti,ab. 1939

18 Bupropion/ 3235

19 (Bupropion or Zyban OR Amfebutamone).ti,ab. 4674

20 12 or 13 or 14 or 15 or 16 or 17 or 18 or 19 40785

21 11 or 20 180369

22 (surger* or surgical or operation or operativ* or an?esthesia or "post-operat*" or postoperat* or "pre-operat*" or preoperat* or "peri-operat*" or perioperat* or "post-surg*" or postsurg* or "pre-surg*" or presurg* or "peri-surg*" or perisurg*).ti,ab. 2730098

23	exp Postoperative complication/	595918
24	exp Preoperative care/	72452
25	22 or 23 or 24	2967693
26	21 and 25	8458
27	exp animals/ not exp humans/	5024857
28	26 not 27	8213
