# Supplementary material for: Protocol for a systematic review and meta-analysis of tobacco-cessation interventions delivered perioperatively
Source: BMJ Open. 2023 Sep 15;13(9):e067722. doi: 10.1136/bmjopen-2022-067722 (PMC10510911; doi:10.1136/bmjopen-2022-067722)
Supplement: Author's manuscript [file bmjopen-2022-067722.draft_revisions.pdf]

BMJ Open is committed to open peer review. As part of this commitment we make the peer review history of every article we publish publicly available.

When an article is published we post the peer reviewers' comments and the authors' responses online. We also post the versions of the paper that were used during peer review. These are the versions that the peer review comments apply to.

The versions of the paper that follow are the versions that were submitted during the peer review process. They are not the versions of record or the final published versions. They should not be cited or distributed as the published version of this manuscript.

BMJ Open is an open access journal and the full, final, typeset and author-corrected version of record of the manuscript is available on our site with no access controls, subscription charges or pay-per-view fees (http://bmjopen.bmj.com).

If you have any questions on BMJ Open's open peer review process please email info.bmjopen@bmj.com

BMJ Open

# Protocol for a systematic review and meta-analysis of tobacco-cessation interventions delivered perioperatively

| | |
|---|---|
| Journal: | *BMJ Open* |
| Manuscript ID | bmjopen-2022-067722 |
| Article Type: | Protocol |
| Date Submitted by the Author: | 30-Aug-2022 |
| Complete List of Authors: | Harrogate, Suzanne; University of Bristol School of Medical Sciences, Elizabeth Blackwell Institute; University Hospitals Bristol and Weston NHS Foundation Trust, Department of Anaesthesia<br>Barnes, Jonathan; University Hospitals Bristol and Weston NHS Foundation Trust, Department of Anaesthesia<br>Gupta, Swati; University Hospitals Bristol and Weston NHS Foundation Trust, Department of Anaesthesia<br>Rudd, Sarah; North Bristol NHS Trust<br>Banerjee, Trisha; Leicester Medical School<br>Thomas, Kyla; University of Bristol, School of Social and Community Medicine<br>Hinchliffe, Robert; University of Bristol, Population Health Sciences; North Bristol NHS Trust<br>Mouton, Ronelle; University of Bristol, Population Health Sciences; North Bristol NHS Trust |
| Keywords: | ANAESTHETICS, PUBLIC HEALTH, SURGERY, Adult anaesthesia < ANAESTHETICS |
| | |

SCHOLARONE™
Manuscripts

# Protocol for a systematic review and meta-analysis of tobacco-cessation interventions delivered perioperatively


## Authors

Suzanne Harrogate 1,2

Jonathan Barnes 2

Swati Gupta 2

Sarah Rudd 3

Trisha Banerjee 4

Kyla Thomas 5

Robert Hinchliffe 3,5

Ronelle Mouton 3,5

## Institutions

1. Elizabeth Blackwell Institute, University of Bristol
2. University Hospitals Bristol and Weston NHS Foundation Trust, Bristol, UK
3. North Bristol NHS Trust, Bristol, UK
4. University of Leicester, Leicester, UK
5. University of Bristol, Bristol, UK

## Correspondence to

Suzanne Harrogate

suzanne.harrogate@nhs.net

University Hospitals Bristol and Weston

Bristol Royal Infirmary

Upper Maudlin Street

BS2 8HW


For peer review only

## Category

Protocol

## Funding statement

This study was supported by the North Bristol NHS Trust and the University of Bristol. SH is supported by the Elizabeth Blackwell Institute for Health Research, University of Bristol and the Wellcome Trust Institutional Strategic Support Fund, WT ISSF3 grant number 204813/Z/16/Z. RM is supported by the North Bristol Vascular Surgery Charitable Trust. KT is funded by a National Institute for Health and Care Research (NIHR) Advanced Fellowship: PDF-2017-10-068. The views expressed in this publication are those of the authors and not necessarily those of the NHS, the NIHR, the Wellcome Trust or the University of Bristol.

## Data Availability Statement

No additional data available

## Abstract

**Introduction**

Tobacco smoking is associated with a substantially increased risk of perioperative complications. The perioperative period is an opportunity to introduce tobacco-cessation strategies. A previous systematic review provided evidence that perioperative interventions increase short-term abstinence and may reduce postoperative complications. The evidence base has since expanded, with the subsequent publication of numerous randomised studies. This protocol outlines a systematic review examining the impact of perioperative tobacco-cessation interventions on successful abstinence from tobacco smoking, and on the incidence of perioperative complications.

**Methods and Analysis**

A systematic search of the literature will be run across EMBASE (Ovid), Medline (Ovid), CINAHL (Ebsco) and PsychInfo (ProQuest), from inception to present, using text words and subject headings. Randomised controlled trials (RCTs) published in English, examining adults in the perioperative period, and reporting the outcomes from tobacco-cessation interventions will be included.

Abstract screening and data extraction will be performed by five reviewers. Each abstract will be screened by two blinded reviewers, with discrepancies resolved by group consensus. The primary outcome will be point prevalence abstinence (PPA) from tobacco-use at the time of surgery. Secondary outcomes are prolonged abstinence from tobacco-use at 6 months and 12 months, and postoperative complications. Any other reported outcomes will be documented in the descriptive analysis. The review will also describe details of the investigated perioperative tobacco-cessation interventions. If sufficient studies report relevant data, meta-analysis of the primary and secondary outcomes will be undertaken. Results will be reported according to the Preferred Reporting items for Systematic Reviews and Meta-Analyses (PRISMA) statement [1].

**Ethics and dissemination**

No ethical approval is required. Results will be disseminated by open-access, peer-reviewed journal publication, and conference presentations. Results will underpin future work to modify

For peer review only

perioperative tobacco-cessation interventions to enhance engagement and accessibility, and to develop trials aiming to facilitate abstinence from tobacco-use in patients presenting for surgery.

## Strengths and limitations of this study

1. This review will provide an up-to-date summary of the evidence for perioperative tobacco-cessation strategies in surgical patients

2. We will describe perioperative tobacco-cessation regimes, methods of assessing abstinence from tobacco-use, and other outcomes assessed in the literature

3. Language restriction to English might exclude additional studies published in other languages.

4. Possible heterogeneity relating to definitions, nomenclature, interventions, and types of outcome measures could limit conclusions.

## Introduction

**Rationale**

Tobacco is a legally regulated, but widely available, recreational substance. Tobacco smoking kills more people than any other addiction, and it is the underlying cause responsible for the greatest number of attributable deaths worldwide [2]. Half of all smokers die prematurely from a cause related to their smoking [2]. 14.1% of adults in the UK (approximately 6.9 million people) smoke tobacco but < 5% are provided with medical support to stop [3]. Smoking related diseases and complications cost the NHS £2billion/year and are the leading cause of health inequalities in England, accounting for over half the difference in the risk of premature death between social classes [4].

An estimated 312.9 million operations are performed annually worldwide [5]. Patients who smoke tobacco face a significantly increased risk of death during the perioperative period and up to one year after their operation. Perioperative morbidity is also significantly higher in tobacco smokers, with a greater incidence of serious complications including surgical site infection, pulmonary complications such as pneumonia and prolonged ventilatory wean, and major adverse cardiac events [6, 7]. Certain surgical populations have a very high prevalence of smoking; data from the National Vascular Registry suggests that a third of all patients undergoing vascular surgery are active tobacco smokers [8]. Smoking-cessation services are also underutilised by the vascular patient group [9].

Preparation for surgery represents a unique opportunity and a so called "teachable moment": a time at which individuals are motivated to change their behaviour. Major surgery has previously been associated with a twofold increase in successful smoking-cessation [10]. The National Institute for Health and Care Excellence (NICE) recommends that health and social care professionals ask their patients about smoking “at every opportunity” and “if they smoke, advise them to stop smoking in a way that is sensitive to their preferences and needs…Explain how stop-smoking support can help” [11].

A 2014 systematic review [12] of thirteen studies examining the effect of preoperative smoking-cessation interventions on abstinence from smoking and on the incidence of postoperative complications found some evidence that preoperative interventions providing behavioural support

For peer review only

and offering nicotine replacement therapy (NRT) increase short-term smoking cessation and may reduce postoperative morbidity. The evidence base has since expanded, with the subsequent publication of numerous randomised controlled trials exploring perioperative interventions aiming to reduce tobacco smoking. NICE guidance was also recently updated [11] to emphasise that although the patient is addicted to nicotine, the harms of smoking are overwhelmingly due to other components derived from tobacco. They advise a treatment strategy that targets a reduction in tobacco-use, rather than nicotine addiction, for example through the prescription of medicinally licensed nicotine-containing products known as nicotine replacement therapy (NRT). As there is no association between NRT and surgical complications, perioperative NRT is considered to be less harmful than perioperative smoking [13].

This protocol outlines an updated systematic review and meta-analysis of the evidence for perioperative tobacco-cessation interventions in surgical patients.

For peer review only

# Aim

The aim of this systematic review is to assess the impact of perioperative interventions on tobacco-cessation and perioperative complications in surgical patients.

**Specific objectives**

1. To identify the surgical populations in which RCTs have been performed comparing the impact of perioperative tobacco-cessation interventions on the prevalence of tobacco-cessation and on postoperative complications.

2. Describe specific tobacco-cessation interventions that have been evaluated perioperatively in randomised controlled trials.

3. Describe the specific outcomes assessed in randomised controlled trials of perioperative tobacco-cessation interventions

4. Describe the specific methods used to assess abstinence from tobacco-use perioperatively in randomised controlled trials

5. To produce an up-to-date examination of the evidence for the impact of perioperative tobacco-cessation interventions on abstinence from tobacco-use at the time of surgery and longer-term, and on the incidence of perioperative complications

# Methods

This protocol follows the statement and checklist of Preferred Reporting Items for Systematic reviews and Meta-Analyses Protocols (PRISMA-P) Statement [14]. The protocol has been registered with the International Prospective Register of Systematic Reviews (PROSPERO) [15] and will be updated if amendments are required.

**Information sources and search strategy**

The search strategy (Appendix 1) has been developed in collaboration with an experienced academic librarian. The following databases: EMBASE (Ovid), Medline (Ovid), CINAHL (Ebsco) and PsychInfo (ProQuest), will be searched from inception to present. The search will include text words and subject headings relating to "smoking-cessation", "tobacco-cessation", and "perioperative". The

reference lists of included papers and relevant grey literature will be hand searched for additional papers. The search results will be limited to English language papers.

**Study selection, inclusion, and exclusion criteria**

This systematic review will include randomised controlled trials (RCTs); cohort, case-control and other non-randomised studies will be excluded. RCTs published in English, in any healthcare setting worldwide, will be examined; no limits will be placed on the country of study or the type of healthcare setting.

RCTs will be included in the review if they meet the following eligibility criteria:

- **Population**
  Adult patients (aged 18 and above) undergoing any type of elective or urgent surgery who are current tobacco smokers
- **Intervention**
  Any tobacco-cessation intervention, used individually or in combination, delivered during the perioperative period
- **Comparator**
  Usual care
- **Outcomes**
  The primary outcome will be point prevalence abstinence (PPA) from tobacco-use at the time of surgery. The secondary outcomes are prolonged abstinence from tobacco-use, measured at 6 months and at 12 months, and postoperative complications. Any other reported outcomes (for example, clinical outcomes, patient-reported outcomes, economic outcomes) will be documented as part of the descriptive analysis.

Perioperative will be defined as the time-period "from the moment of contemplation of surgery until full recovery" [16]. Smoking, or tobacco smoking, will be defined as inhalational tobacco-use (including smoking cigarettes, cigars, cigarillos, pipe tobacco, and other forms of inhalational tobacco as described by the World Health Organisation) [17]. Nicotine replacement therapy (NRT), abstinence, point prevalence abstinence (PPA), and prolonged abstinence will be defined according to the Cochrane Tobacco Addiction Group (TAG) [18]. Abstinence assessed by all methods will be included, whether biochemically verified or self-reported, and methods used by the included studies to assess abstinence will be documented. Perioperative complications will be defined as a composite outcome, and as wound-related, cardiorespiratory, and other complications requiring treatment as described in the 2014 Cochrane Review [12].

**Study records**

*Data management*

The software EndNote (Clarivate 2022) [19] will be used for reference management, initial de-duplication, and screening for language. The software Rayann (2016) [20] will be used for subsequent citation management and screening. Data will be managed using Microsoft Excel (Microsoft Corporation 2018) [21].

*Selection process*

Title and abstracts of all citations identified by the search strategy will be divided and screened for eligibility by five reviewers, with each abstract cross-checked by a second independent, blinded reviewer. After removing blinding, disagreements will be resolved by group discussion and

For peer review only

consensus. This will be followed by independent full text screening of the selected studies by at least two blinded reviewers. Again, disagreements will be resolved by group discussion and consensus.

*Data collection process*

Data will be extracted by two reviewers using a standardised data extraction form and checked by co-reviewers. This form will initially be trialled in four studies, and any necessary changes made after discussion between authors prior to further data collection. Disagreements or discrepancies relating to data extraction will be resolved by group discussion and consensus. Study authors will be contacted directly to request clarification if data is missing or unclear.

*Data Items*

Data to be extracted will include:

Demographic data:

- Publication details (authors, year of publication and country of study)
- Participant demographics (sex, age, number, ASA classification, surgery type and urgency)
- Study details (inclusion/exclusion criteria, number of patients in control and intervention groups)
- Description of tobacco-cessation intervention protocol(s) (behavioural, pharmacotherapy; multimodal; including drugs used, doses, timing relative to surgery, duration and follow-up)
- Description of control protocol
- Definition of abstinence, method of assessing abstinence (patient reported or biochemically validated, time points)

Outcomes data:

- Point prevalence abstinence from tobacco-use at the time of surgery.
- Prolonged abstinence from tobacco-use, measured at 6 months and at 12 months
- Complications including mortality, overall morbidity, length of stay, ICU admission, surgical site infection, delirium, AKI, major adverse cardiac events, postoperative pulmonary complications
- Any additional specific outcomes reported (for example: clinical outcomes, patient-reported outcomes, economic outcomes)

**Evaluation of risk of bias?**

Risk of bias at the study level will be assessed using the Cochrane Risk of Bias 2 (RoB2) tool [22]. At least two reviewers will assess bias in the following domains:

1. Risk of bias arising from the randomisation process.
2. Risk of bias due to deviations from the intended interventions (effects of assignment to intervention and effect of adhering to intervention).
3. Risk of bias due to missing outcome data.
4. Risk of bias in measurement of the outcome.
5. Risk of bias in selection of the reported result.

For peer review only

Trial quality and overall risk of bias (low risk, high risk and some concern) will be determined for each study.

**Data Analysis**

A Preferred Reporting Items for Systematic Reviews and Meta-Analyses (PRISMA) [1] flow chart of search and study selection will be reported and excluded studies will be presented, including reasons for study exclusion. Extracted study data will be presented in tables.

A narrative description of the included studies will be provided. This will include tables summarising study details, including trial design, participant characteristics and reported outcome measures. This will allow comparison of methodology and outcome reporting between different studies. Secondary outcome measures will be tabulated and a narrative description provided.

Meta-analysis of the primary outcome will be undertaken using Stata (StataCorp 2021, College Station, Texas, USA) [23]. The prevalence of tobacco-cessation will be summarised using s rate ratios (with associated 95% confidence intervals) for individual studies and combined using random effect meta-analysis, as recommended by the Cochrane Tobacco Addiction Group (TAG) [12]. If sufficient studies report the secondary outcome of postoperative complications, data will be summarised using mean differences (with associated 95% confidence intervals) for individual studies and by fixed effects meta-analysis. If sufficient data are available, subgroup analysis of studies reporting on patients presenting for vascular surgery and cardiothoracic surgery, presence of co-morbidities, heavy smokers (>20 cigarettes per day) and users of smokeless tobacco will be performed. Forest plots will be produced.

**Assessment of heterogeneity**

Between-study heterogeneity will be assessed using the I2 statistic and random-effects estimates presented if significant heterogeneity is present. A funnel plot will be used to assess publication bias and an influence analysis will be performed by the leave-one-out method. Sensitivity analyses will be undertaken where issues are identified during the review process. Potential analyses include participant characteristics (e.g. varying the lower age limit) and characteristics of comparators (e.g. placebo or usual care).

**Consultation Phase**

Senior members of the review team will guide additional data extraction from specific relevant papers as appropriate.

**Patient and public involvement**

Neither patients nor members of the public were involved in the development of the research question and study design. No new information from patients or members of the public is required to complete this systematic review and meta-analysis.

## Ethics and dissemination:

No ethical approval is required for systematic reviews. The results will be disseminated by presentation at conferences and publication in an open-access peer-reviewed journal. This work will be used to inform a qualitative study into perioperative tobacco-cessation. Result will form the basis for further work aiming to modify and improve perioperative tobacco-cessation interventions to

For peer review only

enhance engagement and accessibility, and to develop future trials aiming ultimately to facilitate abstinence from tobacco-use in patients presenting for surgery.

## Author contributions:

All authors contributed to the concept and design of the systematic review for which this protocol has been written. SH and RM wrote the first draft of the manuscript. JB, RH, SR and KT contributed to subsequent drafts. All authors reviewed and approved the final manuscript before submission.

## Competing interests statement.

The authors have no competing interests to declare.

## Licence Statement

I, the Submitting Author has the right to grant and does grant on behalf of all authors of the Work (as defined in the below author licence), an exclusive licence and/or a non-exclusive licence for contributions from authors who are: i) UK Crown employees; ii) where BMJ has agreed a CC-BY licence shall apply, and/or iii) in accordance with the terms applicable for US Federal Government officers or employees acting as part of their official duties; on a worldwide, perpetual, irrevocable, royalty-free basis to BMJ Publishing Group Ltd ("BMJ") its licensees and where the relevant Journal is co-owned by BMJ to the co-owners of the Journal, to publish the Work in BMJ Open and any other BMJ products and to exploit all rights, as set out in our licence.

The Submitting Author accepts and understands that any supply made under these terms is made by BMJ to the Submitting Author unless you are acting as an employee on behalf of your employer or a postgraduate student of an affiliated institution which is paying any applicable article publishing charge ("APC") for Open Access articles. Where the Submitting Author wishes to make the Work available on an Open Access basis (and intends to pay the relevant APC), the terms of reuse of such Open Access shall be governed by a Creative Commons licence – details of these licences and which Creative Commons licence will apply to this Work are set out in our licence referred to above.

## Word Count:

2782

For peer review only

# Full references


1. Page MJ, et al. *The PRISMA 2020 statement: an updated guideline for reporting systematic reviews.* BMJ, 2021: p. 71.
2. World Health Organisation. *WHO report on the global tobacco epidemic, 2017: monitoring tobacco use and prevention policies*. 2017. World Health Organization, Geneva.
3. Windsor-Shellard B. *Adult smoking habits in the UK - Office for National Statistics*. 2022, Office for National Statistics Annual Population Survey.
4. ASH. *ASH Briefing: Health inequalities and smoking*. 2016. Action on Smoking and Health, United Kingdom.
5. Weiser TG, et al. *Estimate of the global volume of surgery in 2012: an assessment supporting improved health outcomes.* The Lancet, 2015. **385**: p. S11.
6. Turan A, et al., *Smoking and Perioperative Outcomes.* Anesthesiology, 2011. **114**(4): p. 837-846.
7. Hawn MT, et al. *The Attributable Risk of Smoking on Surgical Complications.* Annals of Surgery, 2011. **254**(6): p. 914-920.
8. *National Vascular Registry: 2021 Annual Report*. 2021. Vascular Services Quality Imporvement Programme, Royal College of Surgeons of England, UK.
9. Patel KK et al. *Underutilization of Evidence-Based Smoking Cessation Support Strategies Despite High Smoking Addiction Burden in Peripheral Artery Disease Specialty Care: Insights from the International PORTRAIT Registry.* Journal of the American Heart Association, 2018. **7**(20).
10. Shi Y and Warner DO *Surgery as a Teachable Moment for Smoking Cessation.* Anesthesiology, 2010. **112**(1): p. 102-107.
11. National Institute for, Health and Care Excellence. (2021). *Tobacco: preventing uptake, promoting quitting and treating dependence*. [NICE Guideline No. 209] Available from: https://www.nice.org.uk/guidance/ng209
12. Thomsen T, Villebro N, and Møller AM. *Interventions for preoperative smoking cessation.* Cochrane Database of Systematic Reviews, 2014.
13. Sørensen LT. *Wound Healing and Infection in Surgery: The Pathophysiological Impact of Smoking, Smoking Cessation, and Nicotine Replacement Therapy: A Systematic Review.* Annals of Surgery, 2012. **255**(6): p. 1069-1079.
14. Moher D, et al., *Preferred reporting items for systematic review and meta-analysis protocols (PRISMA-P) 2015 statement.* Systematic Reviews, 2015. **4**(1): p. 1.
15. Harrogate S, et al. *PROSPERO: International prospective register of systematic reviews. 2022. CRD42022351992.* Available from: https://www.crd.york.ac.uk/prospero/display_record.php?ID=CRD42022351992. 2022.
16. The Centre for Perioperative Care. *The Case for Perioperative Care: Describing the Need*. 2019 [cited 2022; Available from: https://www.cpoc.org.uk/node/396.
17. World Health Organisation. *Fact Sheet: Tobacco*. 2022, World Health Organisation: World Health Organisation Fact Sheets.
18. Cochrane Tobacco Addiction Group (TAG), *Glossary of Terms*. (2022) [cited 2022; Available from: https://tobacco.cochrane.org/resources/glossary]
19. *EndNote*. 2013, Clarivate: Philadelphia, PA.
20. Ouzzani MH, Hossam. Fedorowicz Z, Elmagarmid A. *Rayyan — a web and mobile app for systematic reviews.* 2016.
21. *Microsoft Excel*. 2018, Microsoft Corporation.
22. Sterne JAC, et al. *RoB 2: a revised tool for assessing risk of bias in randomised trials.* BMJ, 2019: p. l4898.
23. *Stata Statistical Software*, StataCorp. Editor. 2021: College Station, TX.


For peer review only

## Appendix 1: Search strategy

**The search has been developed with the help of a librarian. A draft of the search is as follows:**

Ovid MEDLINE(R) ALL <1946 to July 2022>

1 smoke*.ti,ab. 147122

2 "Tobacco Use Disorder"/ 12064

3 exp Smoking/ 158059

4 Tobacco/ or exp Tobacco Products/ 42941

5 tobacco.ti,ab. 110972

6 exp Smoking Devices/ 15555

7 (cigarette* or "e-cigarette*" or "electronic cigarette*" or vape* or vap?ing or cigar?).ti,ab. 80944

8 exp pipe smoking/ or exp tobacco smoking/ or vaping/ 153721

9 1 or 2 or 3 or 4 or 5 or 6 or 7 or 8 319299

10 (reduc* or quit* or stop* or abstin* or abstain* or cessat* or prevent* or consum* or program or intervention or "cut* down" or "cut-down" or "cut back" or "cut-back" or "cold turkey" or control* or schedul*).ti,ab. 9267831

11 9 and 10 167244

12 Smoking Cessation/ 31670

13 "Tobacco Use Cessation"/ 1386

14 exp "Tobacco Use Cessation Devices"/ 2355

15 ((Nicotine or tobacco) adj5 (replacement or cessation or patch* or "chewing gum" or inhalator* or tablet* or "oral strip*" or lozenge* or spray*)).ti,ab. 10237

16 Varenicline/ 1478

17 (Varenicline or champix).ti,ab. 1939

18 Bupropion/ 3235

19 (Bupropion or Zyban OR Amfebutamone).ti,ab. 4674

20 12 or 13 or 14 or 15 or 16 or 17 or 18 or 19 40785

21 11 or 20 180369

22 (surger* or surgical or operation or operativ* or an?esthesia or "post-operat*" or postoperat* or "pre-operat*" or preoperat* or "peri-operat*" or perioperat* or "post-surg*" or postsurg* or "pre-surg*" or presurg* or "peri-surg*" or perisurg*).ti,ab. 2730098

For peer review only

| | | |
|---|---|---|
| 23 | exp Postoperative complication/ | 595918 |
| 24 | exp Preoperative care/ | 72452 |
| 25 | 22 or 23 or 24 | 2967693 |
| 26 | 21 and 25 | 8458 |
| 27 | exp animals/ not exp humans/ | 5024857 |
| 28 | 26 not 27 | 8213 |

For peer review only

1

# PRISMA-P 2015 Checklist for "Protocol for a systematic review and meta-analysis of tobacco-cessation interventions delivered perioperatively"

This checklist has been adapted for use with protocol submissions to *Systematic Reviews* from Table 3 in Moher D et al: Preferred reporting items for systematic review and meta-analysis protocols (PRISMA-P) 2015 statement. *Systematic Reviews* 2015 **4**:1

| Section/topic | # | Checklist item | Information reported | | Line number(s) |
|---|---|---|---|---|---|
| | | | Yes | No | |
| **ADMINISTRATIVE INFORMATION** | | | | | |
| **Title** | | | | | |
| | | | | | |
| Identification | 1a | Identify the report as a protocol of a systematic review | ☒ | ☐ | 1, 41, 115 |
| Update | 1b | If the protocol is for an update of a previous systematic review, identify as such | ☐ | ☒ | N/A |
| **Registration** | 2 | If registered, provide the name of the registry (e.g., PROSPERO) and registration number in the Abstract | ☒ | ☐ | 46, 136 |
| **Authors** | | | | | |
| Contact | 3a | Provide name, institutional affiliation, and e-mail address of all protocol authors; provide physical mailing address of corresponding author | ☒ | ☐ | 4-24 |
| Contributions | 3b | Describe contributions of protocol authors and identify the guarantor of the review | ☒ | ☐ | 265 |
| **Amendments** | 4 | If the protocol represents an amendment of a previously completed or published protocol, identify as such and list changes; otherwise, state plan for documenting important protocol amendments | ☐ | ☒ | N/A |
| **Support** | | | | | |
| Sources | 5a | Indicate sources of financial or other support for the review | ☒ | ☐ | 27 |
| Sponsor | 5b | Provide name for the review funder and/or sponsor | ☒ | ☐ | 27 |
| Role of sponsor/funder | 5c | Describe roles of funder(s), sponsor(s), and/or institution(s), if any, in developing the protocol | ☐ | ☒ | N/A |
| **INTRODUCTION** | | | | | |
| **Rationale** | 6 | Describe the rationale for the review in the context of what is already known | ☒ | ☐ | 79-115 |

BioMed Central The Open Access Publisher

| Section/topic | # | Checklist item | Information reported | | Line number(s) |
|---|---|---|---|---|---|
| | | | Yes | No | |
| **Objectives** | 7 | Provide an explicit statement of the question(s) the review will address with reference to participants, interventions, comparators, and outcomes (PICO) | ☒ | ☐ | 119, 151 |
| **METHODS** | | | | | |
| **Eligibility criteria** | 8 | Specify the study characteristics (e.g., PICO, study design, setting, time frame) and report characteristics (e.g., years considered, language, publication status) to be used as criteria for eligibility for the review | ☒ | ☐ | 147-175 |
| **Information sources** | 9 | Describe all intended information sources (e.g., electronic databases, contact with study authors, trial registers, or other grey literature sources) with planned dates of coverage | ☒ | ☐ | 140-145 |
| **Search strategy** | 10 | Present draft of search strategy to be used for at least one electronic database, including planned limits, such that it could be repeated | ☒ | ☐ | Appendix |
| ***STUDY RECORDS*** | | | | | |
| Data management | 11a | Describe the mechanism(s) that will be used to manage records and data throughout the review | ☒ | ☐ | 177-181 |
| Selection process | 11b | State the process that will be used for selecting studies (e.g., two independent reviewers) through each phase of the review (i.e., screening, eligibility, and inclusion in meta-analysis) | ☒ | ☐ | 182-187 |
| Data collection process | 11c | Describe planned method of extracting data from reports (e.g., piloting forms, done independently, in duplicate), any processes for obtaining and confirming data from investigators | ☒ | ☐ | 188-193 |
| **Data items** | 12 | List and define all variables for which data will be sought (e.g., PICO items, funding sources), any pre-planned data assumptions and simplifications | ☒ | ☐ | 194-213 |
| **Outcomes and prioritization** | 13 | List and define all outcomes for which data will be sought, including prioritization of main and additional outcomes, with rationale | ☒ | ☐ | 206-213 |
| **Risk of bias in individual studies** | 14 | Describe anticipated methods for assessing risk of bias of individual studies, including whether this will be done at the outcome or study level, or both; state how this information will be used in data synthesis | ☒ | ☐ | 214-224 |
| ***DATA*** | | | | | |
| **Synthesis** | 15a | Describe criteria under which study data will be quantitatively synthesized | ☒ | ☐ | 226-242 |
| | 15b | If data are appropriate for quantitative synthesis, describe planned summary measures, methods of handling data, and methods of combining data from studies, including any planned exploration of consistency (e.g., $I^2$, Kendall's tau) | ☒ | ☐ | 244 |
| | 15c | Describe any proposed additional analyses (e.g., sensitivity or subgroup analyses, meta- | ☒ | ☐ | 233 |

For peer review only

BioMed Central The Open Access Publisher

| Section/topic | # | Checklist item | Information reported Yes | No | Line number(s) |
|---|---|---|---|---|---|
| | | regression) | | | |
| | 15d | If quantitative synthesis is not appropriate, describe the type of summary planned | ☒ | ☐ | 229 |
| **Meta-bias(es)** | 16 | Specify any planned assessment of meta-bias(es) (e.g., publication bias across studies, selective reporting within studies) | ☒ | ☐ | 233 |
| **Confidence in cumulative evidence** | 17 | Describe how the strength of the body of evidence will be assessed (e.g., GRADE) | ☒ | ☐ | 223 |

For peer review only



BioMed Central The Open Access Publisher

BMJ Open

# Protocol for a systematic review and meta-analysis of tobacco-cessation interventions delivered perioperatively

| | |
|---|---|
| Journal: | *BMJ Open* |
| Manuscript ID | bmjopen-2022-067722.R1 |
| Article Type: | Protocol |
| Date Submitted by the Author: | 09-Jun-2023 |
| Complete List of Authors: | Harrogate, Suzanne; University of Bristol School of Medical Sciences, Elizabeth Blackwell Institute; University Hospitals Bristol and Weston NHS Foundation Trust, Department of Anaesthesia<br>Barnes, Jonathan; University Hospitals Bristol and Weston NHS Foundation Trust, Department of Anaesthesia<br>Gupta, Swati; University Hospitals Bristol and Weston NHS Foundation Trust, Department of Anaesthesia<br>Rudd, Sarah; North Bristol NHS Trust<br>Banerjee, Trisha; Leicester Medical School<br>Thomas, Kyla; University of Bristol, School of Social and Community Medicine<br>Hinchliffe, Robert; University of Bristol, Population Health Sciences; North Bristol NHS Trust<br>Mouton, Ronelle; University of Bristol, Population Health Sciences; North Bristol NHS Trust |
| <b>Primary Subject Heading</b>: | Anaesthesia |
| Secondary Subject Heading: | Addiction, Smoking and tobacco |
| Keywords: | ANAESTHETICS, PUBLIC HEALTH, SURGERY, Adult anaesthesia < ANAESTHETICS |
| | |

SCHOLARONE™
Manuscripts

# Protocol for a systematic review and meta-analysis of tobacco-cessation interventions delivered perioperatively


## Authors

Suzanne Harrogate 1,2

Jonathan Barnes 2

Swati Gupta 2

Sarah Rudd 3

Trisha Banerjee 4

Kyla Thomas 5

Robert Hinchliffe 3,5

Ronelle Mouton 3,5

## Institutions

1. Elizabeth Blackwell Institute, University of Bristol
2. University Hospitals Bristol and Weston NHS Foundation Trust, Bristol, UK
3. North Bristol NHS Trust, Bristol, UK
4. University of Leicester, Leicester, UK
5. University of Bristol, Bristol, UK

## Correspondence to

Dr Suzanne Harrogate

suzanne.harrogate@nhs.net

University Hospitals Bristol and Weston

Bristol Royal Infirmary

Upper Maudlin Street

BS2 8HW


For peer review only

## Category

Protocol

## Funding statement

This study was supported by the North Bristol NHS Trust and the University of Bristol. SH is supported by the Elizabeth Blackwell Institute for Health Research, University of Bristol and the Wellcome Trust Institutional Strategic Support Fund, WT ISSF3 grant number 204813/Z/16/Z. RM is supported by the North Bristol Vascular Surgery Charitable Trust. KT is funded by a National Institute for Health and Care Research (NIHR) Advanced Fellowship: PDF-2017-10-068. The views expressed in this publication are those of the authors and not necessarily those of the NHS, the NIHR, the Wellcome Trust or the University of Bristol.

## Data Availability Statement

No additional data available

## Abstract

**Introduction**

Tobacco smoking is associated with a substantially increased risk of perioperative complications. The perioperative period is an opportunity to introduce tobacco-cessation strategies. A previous systematic review provided evidence that perioperative interventions increase short-term abstinence and may reduce postoperative complications. The evidence base has since expanded, with the subsequent publication of numerous randomised studies. This protocol outlines a systematic review examining the impact of perioperative tobacco-cessation interventions on successful abstinence from tobacco smoking, and on the incidence of perioperative complications.

**Methods and Analysis**

A systematic search of the literature will be run across EMBASE (Ovid), Medline (Ovid), CINAHL (Ebsco) and PsychInfo (ProQuest), from inception to present, using text words and subject headings. Randomised controlled trials (RCTs) published in English, examining adults in the perioperative period, and reporting the outcomes from tobacco-cessation interventions will be included.

Abstract screening and data extraction will be performed by five reviewers. Each abstract will be screened by two blinded reviewers, with discrepancies resolved by group consensus. The primary outcome will be point prevalence abstinence (PPA) from tobacco-use at the time of surgery. Secondary outcomes are prolonged abstinence from tobacco-use at 6 months and 12 months, and postoperative complications. Any other reported outcomes will be documented in the descriptive analysis. The review will also describe details of the investigated perioperative tobacco-cessation interventions. If sufficient studies report relevant data, meta-analysis of the primary and secondary outcomes will be undertaken. Results will be reported according to the Preferred Reporting items for Systematic Reviews and Meta-Analyses (PRISMA) statement.

**Ethics and dissemination**

No ethical approval is required. Results will be disseminated by open-access, peer-reviewed journal publication, and conference presentations. Results will underpin future work to modify

For peer review only

perioperative tobacco-cessation interventions to enhance engagement and accessibility, and to develop trials aiming to facilitate abstinence from tobacco-use in patients presenting for surgery.

## Strengths and limitations of this study

1. This systematic review will be based on a comprehensive search strategy including randomised controlled trials.

2. This systematic review will go beyond those previously published in this context by including smoking cessation interventions which take place at any point during the perioperative pathway, increasing the real-world relevance of the findings.

3. Language restriction to English may exclude additional studies published in other languages.

4. There may be some effective smoking cessation interventions for which there is scant data in perioperative settings.

## Introduction

Tobacco is a legally regulated, but widely available, recreational substance. Tobacco smoking kills more people than any other addiction, and it is the agent responsible for the greatest number of attributable deaths worldwide [1]. Half of all smokers die prematurely from a cause related to their smoking [1]. 14.1% of adults in the UK (approximately 6.9 million people) smoke tobacco but < 5% are provided with medical support to stop [2]. Smoking related diseases and complications cost the NHS £2billion/year and are the leading cause of health inequalities in England, accounting for over half the difference in the risk of premature death between social classes [3].

An estimated 312.9 million operations are performed annually worldwide [4]. Patients who smoke tobacco face a significantly increased risk of death during the perioperative period and up to one year after their operation. Perioperative morbidity is also significantly higher in tobacco smokers, with a greater incidence of serious complications including surgical site infection, pulmonary complications such as pneumonia and prolonged ventilatory wean, and major adverse cardiac events [5, 6]. Certain surgical populations have a very high prevalence of smoking; data from the National Vascular Registry suggests that a third of all patients undergoing vascular surgery actively smoke tobacco [7]. Smoking-cessation services are also underutilised in this patient group [8].

Preparation for surgery represents a unique opportunity and a so called "teachable moment": a time at which individuals are motivated to change their behaviour. Major surgery has previously been associated with a twofold increase in successful smoking-cessation [9]. Qualitative studies exploring the patient experience have identified major surgery as a useful cue for motivating patients to quit smoking; abstinence was seen as an “integral part” of the surgery, as patients experienced a focus shift to the operation alongside a reduction in cravings [10]. The National Institute for Health and Care Excellence (NICE) recommends that health and social care professionals ask their patients about smoking “at every opportunity” and “if they smoke, advise them to stop smoking in a way that is sensitive to their preferences and needs…Explain how stop-smoking support can help” [11].

A 2014 systematic review [12] of thirteen studies examining the effect of preoperative smoking-cessation interventions on abstinence from smoking and on the incidence of postoperative complications found evidence that preoperative interventions providing behavioural support and offering nicotine replacement therapy (NRT) increase short-term smoking cessation and may reduce postoperative morbidity. The evidence base has since expanded, with the subsequent publication of

For peer review only

numerous randomised controlled trials exploring perioperative interventions aiming to reduce tobacco smoking. NICE guidance was also recently updated to emphasise that although the patient is addicted to nicotine, the harms of smoking are overwhelmingly due to other components derived from tobacco [11]. A report commissioned by the Office for Health Improvement and Disparities concluded that, when compared to tobacco smoking, the use of alternative nicotine delivery systems such as electronic cigarettes led to significantly lower relative exposure to toxic chemicals, and significantly lower levels of biomarkers associated with the risk of cancer, cardiorespiratory and other health conditions [13]. NICE therefore advise a treatment strategy that targets a reduction in tobacco-use, alongside concurrent treatment of nicotine addiction, for example through the prescription of medicinally licensed nicotine-containing products known as nicotine replacement therapy (NRT). As there is no association between NRT and surgical complications, perioperative NRT is considered to be less harmful than perioperative smoking [14].

This protocol outlines an updated systematic review and meta-analysis of the evidence for perioperative tobacco-cessation interventions in surgical patients.

# Aim

The aim of this systematic review is to assess the impact of perioperative interventions on tobacco-cessation and perioperative complications in surgical patients.

**Specific objectives**

1. To identify the surgical populations in which RCTs have been performed comparing the impact of perioperative tobacco-cessation interventions on the prevalence of tobacco-cessation and on postoperative complications.

2. Describe specific tobacco-cessation interventions that have been evaluated perioperatively in randomised controlled trials.

3. Describe the specific outcomes assessed in randomised controlled trials of perioperative tobacco-cessation interventions

4. Describe the specific methods used to assess abstinence from tobacco-use perioperatively in randomised controlled trials

5. To produce an up-to-date examination of the evidence for the impact of perioperative tobacco-cessation interventions on abstinence from tobacco-use at the time of surgery and longer-term, and on the incidence of perioperative complications

# Methods

This protocol follows the statement and checklist of Preferred Reporting Items for Systematic reviews and Meta-Analyses Protocols (PRISMA-P) Statement [15]. The protocol has been registered with the International Prospective Register of Systematic Reviews (PROSPERO) [16] and will be updated if amendments are required. This study is planned to take place between January and December of 2023.

**Information sources and search strategy**

The search strategy (Appendix 1) has been developed in collaboration with an experienced academic librarian. The following databases: EMBASE (Ovid), Medline (Ovid), CINAHL (Ebsco) and PsychInfo

For peer review only

(ProQuest), will be searched from inception to present. The search will include text words and subject headings relating to "smoking-cessation", "tobacco-cessation", and "perioperative". The reference lists of included papers and relevant grey literature will be hand searched for additional papers. The search results will be limited to English language papers.

**Study selection, inclusion, and exclusion criteria**

This systematic review will include randomised controlled trials (RCTs); cohort, case-control and other non-randomised studies will be excluded. RCTs published in English, in any healthcare setting worldwide, will be examined; no limits will be placed on the country of study or the type of healthcare setting.

RCTs will be included in the review if they meet the following eligibility criteria:

- **Population**
  Adult patients (aged 18 and above) undergoing any type of elective or urgent surgery who are current tobacco smokers.
- **Intervention**
  Any tobacco-cessation intervention, used individually or in combination, delivered during the perioperative period.
- **Comparator**
  Usual care.
- **Outcomes**
  The primary outcome will be point prevalence abstinence (PPA) from tobacco-use at the time of surgery. The secondary outcomes are prolonged abstinence from tobacco-use, measured at 6 months and at 12 months, and postoperative complications. Any other reported outcomes (for example, clinical outcomes, patient-reported outcomes, economic outcomes) will be documented as part of the descriptive analysis.

Perioperative will be defined as the time-period "from the moment of contemplation of surgery until full recovery" [17]. Smoking, or tobacco smoking, will be defined as inhalational tobacco-use (including smoking cigarettes, cigars, cigarillos, pipe tobacco, and other forms of inhalational tobacco as described by the World Health Organisation) [18]. Nicotine replacement therapy (NRT), abstinence, point prevalence abstinence (PPA), and prolonged abstinence will be defined according to the Cochrane Tobacco Addiction Group (TAG) [19]. Abstinence assessed by all methods will be included, whether biochemically verified or self-reported, and methods used by the included studies to assess abstinence will be documented. Perioperative complications will be defined as a composite outcome, and as wound-related, cardiorespiratory, and other complications requiring treatment as described in the 2014 Cochrane Review [12].

**Study records**

*Data management*

The software EndNote (Clarivate 2022) [20] will be used for reference management, initial de-duplication, and screening for language. The software Rayann (Rayann, 2016) [21] will be used for subsequent citation management and screening. Data will be managed using Microsoft Excel (Microsoft Corporation 2018) [22].

*Selection process*

For peer review only

Title and abstracts of all citations identified by the search strategy will be divided and screened for eligibility by five reviewers, with each abstract cross-checked by a second independent, blinded reviewer. After removing blinding, disagreements will be resolved by group discussion and consensus. This will be followed by independent full text screening of the selected studies by at least two blinded reviewers. Again, disagreements will be resolved by group discussion and consensus.

*Data collection process*

Data will be extracted by two reviewers using a standardised data extraction form and checked by co-reviewers. This form will initially be trialled in four studies, and any necessary changes made after discussion between authors prior to further data collection. Disagreements or discrepancies relating to data extraction will be resolved by group discussion and consensus. Study authors will be contacted directly to request clarification if data is missing or unclear.

*Data Items*

Data to be extracted will include:

Demographic data:

- Publication details (authors, year of publication and country of study)
- Participant demographics (sex, age, number, ASA classification, surgery type and urgency)
- Study details (inclusion/exclusion criteria, number of patients in control and intervention groups)
- Description of tobacco-cessation intervention protocol(s) (behavioural, pharmacotherapy, multimodal; mode of delivery [face-to-face, telephone based or digital], drugs and doses used, timing relative to surgery, intervention duration and follow-up)
- Description of intensity of intervention (intensive smoking cessation intervention, short intervention, or other intervention [23])
- Description of control protocol
- Definition of abstinence, method of assessing abstinence (patient reported or biochemically validated, time points)

Outcomes data:

- Point prevalence abstinence from tobacco-use at the time of surgery.
- Prolonged abstinence from tobacco-use, measured at 6 months and at 12 months.
- Reported perioperative complications data (particularly the incidence of any complications, and of wound-related complications).
- Any additional specific outcomes reported (for example: clinical outcomes including mental health, patient-reported outcomes including patient satisfaction, economic outcomes).

**Quality of Evidence and Evaluation of Risk of Bias**

The GRADE system will be used to assess overall quality of evidence [24]. Risk of bias at the study outcomes level will be assessed using the Cochrane Risk of Bias 2 (RoB2) tool [25].

**Data Analysis**

A Preferred Reporting Items for Systematic Reviews and Meta-Analyses (PRISMA) [26] flow chart of search and study selection will be reported and excluded studies will be presented, including reasons for study exclusion. Extracted study data will be presented in tables.

For peer review only

A narrative description of all the included studies will be provided. This will include tables summarising study details including trial design, participant characteristics, and reported outcome measures, as well as Risk of Bias assessments and GRADE assessments for each outcome. This will allow comparison of methodology and outcome reporting between studies.

Meta-analysis of the primary outcome will be undertaken using Review Manager (The Cochrane Collaboration, 2020) [27]. The prevalence of tobacco-cessation will be summarised using rate ratios (with associated 95% confidence intervals) for individual studies and combined using random effect meta-analysis, as recommended by the Cochrane Tobacco Addiction Group (TAG) [11]. Forest plots will be produced. If appropriate, meta-analysis of the secondary smoking cessation and complications outcomes will be undertaken in the same way. If sufficient data are available, the following subgroup analyses will be undertaken: subgroups defined by the intensity of smoking cessation intervention (intensive, short or other) [23], by the timepoint of the intervention in relation to surgery (preoperative only, intraoperative only, postoperative only and both pre- and postoperative), the presence of co-morbidities, and heaviness of smoking. If there are sufficient data, we plan also to also pool the results of studies looking at individual pharmacotherapies (for example, varenicline) only, electronic cigarettes only, and recruiting only patients presenting for vascular surgery and cardiothoracic surgery. Heterogeneity will be assessed using the I2 statistic and random-effects estimates presented assuming significant heterogeneity is present. A funnel plot will be used to assess publication bias and an influence analysis will be performed by the leave-one-out method. Sensitivity analyses will be undertaken where issues are identified during the review process. Potential analyses include participant characteristics (for example: varying the lower age limit) and characteristics of comparators (for example: placebo or usual care).

**Consultation Phase**

Senior members of the review team will guide additional data extraction from specific relevant papers as appropriate.

**Patient and public involvement**

Neither patients nor members of the public were involved in the development of the research question and study design. No new information from patients or members of the public is required to complete this systematic review and meta-analysis.

## Ethics and dissemination:

No ethical approval is required for systematic reviews. The results will be disseminated by presentation at conferences and publication in an open-access peer-reviewed journal. This work will be used to inform a qualitative study into perioperative tobacco-cessation. Result will form the basis for further work aiming to modify and improve perioperative tobacco-cessation interventions to enhance engagement and accessibility, and to develop future trials aiming ultimately to facilitate abstinence from tobacco-use in patients presenting for surgery.

## Author contributions:

All authors contributed to the concept and design of the systematic review for which this protocol has been written. SH and RM wrote the first draft of the manuscript. JB, RH, SR, SG, TB and KT contributed to subsequent drafts. All authors reviewed and approved the final manuscript before submission.

For peer review only

## Competing interests statement.

The authors have no competing interests to declare.

## Licence Statement

I, the Submitting Author has the right to grant and does grant on behalf of all authors of the Work (as defined in the below author licence), an exclusive licence and/or a non-exclusive licence for contributions from authors who are: i) UK Crown employees; ii) where BMJ has agreed a CC-BY licence shall apply, and/or iii) in accordance with the terms applicable for US Federal Government officers or employees acting as part of their official duties; on a worldwide, perpetual, irrevocable, royalty-free basis to BMJ Publishing Group Ltd ("BMJ") its licensees and where the relevant Journal is co-owned by BMJ to the co-owners of the Journal, to publish the Work in BMJ Open and any other BMJ products and to exploit all rights, as set out in our licence.

The Submitting Author accepts and understands that any supply made under these terms is made by BMJ to the Submitting Author unless you are acting as an employee on behalf of your employer or a postgraduate student of an affiliated institution which is paying any applicable article publishing charge ("APC") for Open Access articles. Where the Submitting Author wishes to make the Work available on an Open Access basis (and intends to pay the relevant APC), the terms of reuse of such Open Access shall be governed by a Creative Commons licence – details of these licences and which Creative Commons licence will apply to this Work are set out in our licence referred to above.

## Word Count:

2905

For peer review only

# Full references

1. World Health Organisation. WHO report on the global tobacco epidemic: monitoring tobacco use and prevention policies. 2017. World Health Organization, Geneva.

2. Windsor-Shellard B. Adult smoking habits in the UK - Office for National Statistics Annual Population Survey. Office for National Statistics. 2022.

3. ASH Briefing: Health inequalities and smoking. 2016. Action on Smoking and Health, United Kingdom.

4. Weiser TG, et al. Estimate of the global volume of surgery in 2012: an assessment supporting improved health outcomes. The Lancet. 2015; 385: S11.

5. Turan A, et al. Smoking and Perioperative Outcomes. Anesthesiology, 2011; 114(4): 837-846.

6. Hawn MT, et al. The Attributable Risk of Smoking on Surgical Complications. Annals of Surgery. 2011; 254(6): 914-920.

7. National Vascular Registry: 2021 Annual Report. 2021. Vascular Services Quality Imporvement Programme, Royal College of Surgeons of England, UK.

8. Patel KK et al. Underutilization of Evidence-Based Smoking Cessation Support Strategies Despite High Smoking Addiction Burden in Peripheral Artery Disease Specialty Care: Insights from the International PORTRAIT Registry. Journal of the American Heart Association. 2018; 7(20).

9. Shi Y and Warner DO Surgery as a Teachable Moment for Smoking Cessation. Anesthesiology. 2010; 112(1): 102-107.

10. Lauridsen SV, et al. Smoking and alcohol cessation intervention in relation to radical cystectomy: A qualitative study of cancer patients' experiences. BMC Cancer. 2017; 17(1) 793

11. Tobacco: preventing uptake, promoting quitting and treating dependence. [NICE Guideline No. 209]. 2021. National Institute for Health and Care Excellence. Available from: https://www.nice.org.uk/guidance/ng209

12. Thomsen T, Villebro N, and Møller AM. Interventions for preoperative smoking cessation. Cochrane Database of Systematic Reviews. 2014.

13. McNeill A, et al. Nicotine vaping in England: an evidence update including health risks and perceptions. A report commissioned by the Office for Health Improvement and Disparities. 2022.

14. Sørensen LT. Wound Healing and Infection in Surgery: The Pathophysiological Impact of Smoking, Smoking Cessation, and Nicotine Replacement Therapy: A Systematic Review. Annals of Surgery. 2012; 255(6): 1069-1079.

15. Moher D, et al., Preferred reporting items for systematic review and meta-analysis protocols (PRISMA-P) 2015 statement. Systematic Reviews. 2015; 4(1): 1.

16. Harrogate S, et al. PROSPERO: International prospective register of systematic reviews. 2022. CRD42022351992. Available from: https://www.crd.york.ac.uk/prospero/display_record.php?ID=CRD42022351992. 2022.

For peer review only

17. The Case for Perioperative Care: Describing the Need. 2019. The Centre for Perioperative Care. [cited 2022; Available from: https://www.cpoc.org.uk/node/396.

18. World Health Organisation Fact Sheet: Tobacco. 2022. World Health Organisation

19. Cochrane Tobacco Addiction Group (TAG), Glossary of Terms. (2022) [cited 2022; Available from: https://tobacco.cochrane.org/resources/glossary]

20. EndNote. 2013, Clarivate: Philadelphia, PA.

21. Ouzzani MH, et al. Rayyan — a web and mobile app for systematic reviews. 2016.

22. Microsoft Excel. 2018, Microsoft Corporation.

23. Rasmussen S, et al. Intensive versus short face-to-face smoking cessation interventions: a meta-analysis. European respiratory review : an official journal of the European Respiratory Society. 2022; 31(165) 220063-220063

24. Guyatt G, et al. GRADE guidelines: 11. Making an overall rating of confidence in effect estimates for a single outcome and for all outcomes. Journal of Clinical Epidemiology. 66(2) 151 - 157

25. Sterne JAC, et al. RoB 2: a revised tool for assessing risk of bias in randomised trials. BMJ, 2019: l4898.

26. Page MJ, et al. The PRISMA 2020 statement: an updated guideline for reporting systematic reviews. BMJ. 2021: 71.

27. Review Manager (RevMan) [Computer program]. Version 5.4. The Cochrane Collaboration. 2020.

For peer review only

## Appendix 1: Search strategy

**The search has been developed with the help of a librarian. A draft of the search is as follows:**

Ovid MEDLINE(R) ALL <1946 to July 2022>

1 smoke*.ti,ab. 147122

2 "Tobacco Use Disorder"/ 12064

3 exp Smoking/ 158059

4 Tobacco/ or exp Tobacco Products/ 42941

5 tobacco.ti,ab. 110972

6 exp Smoking Devices/ 15555

7 (cigarette* or "e-cigarette*" or "electronic cigarette*" or vape* or vap?ing or cigar?).ti,ab. 80944

8 exp pipe smoking/ or exp tobacco smoking/ or vaping/ 153721

9 1 or 2 or 3 or 4 or 5 or 6 or 7 or 8 319299

10 (reduc* or quit* or stop* or abstin* or abstain* or cessat* or prevent* or consum* or program or intervention or "cut* down" or "cut-down" or "cut back" or "cut-back" or "cold turkey" or control* or schedul*).ti,ab. 9267831

11 9 and 10 167244

12 Smoking Cessation/ 31670

13 "Tobacco Use Cessation"/ 1386

14 exp "Tobacco Use Cessation Devices"/ 2355

15 ((Nicotine or tobacco) adj5 (replacement or cessation or patch* or "chewing gum" or inhalator* or tablet* or "oral strip*" or lozenge* or spray*)).ti,ab. 10237

16 Varenicline/ 1478

17 (Varenicline or champix).ti,ab. 1939

18 Bupropion/ 3235

19 (Bupropion or Zyban OR Amfebutamone).ti,ab. 4674

20 12 or 13 or 14 or 15 or 16 or 17 or 18 or 19 40785

21 11 or 20 180369

22 (surger* or surgical or operation or operativ* or an?esthesia or "post-operat*" or postoperat* or "pre-operat*" or preoperat* or "peri-operat*" or perioperat* or "post-surg*" or postsurg* or "pre-surg*" or presurg* or "peri-surg*" or perisurg*).ti,ab. 2730098

For peer review only

| | | |
|---|---|---|
| 23 | exp Postoperative complication/ | 595918 |
| 24 | exp Preoperative care/ | 72452 |
| 25 | 22 or 23 or 24 | 2967693 |
| 26 | 21 and 25 | 8458 |
| 27 | exp animals/ not exp humans/ | 5024857 |
| 28 | 26 not 27 | 8213 |

For peer review only

# PRISMA-P 2015 Checklist for "Protocol for a systematic review and meta-analysis of tobacco-cessation interventions delivered perioperatively"

This checklist has been adapted for use with protocol submissions to *Systematic Reviews* from Table 3 in Moher D et al: Preferred reporting items for systematic review and meta-analysis protocols (PRISMA-P) 2015 statement. *Systematic Reviews* 2015 **4**:1

| Section/topic | # | Checklist item | Information reported: Yes | Information reported: No | Line number(s) |
|---|---|---|---|---|---|
| **ADMINISTRATIVE INFORMATION** | | | | | |
| **Title** | | | | | |
| | | | | | |
| Identification | 1a | Identify the report as a protocol of a systematic review | ☒ | ☐ | 1, 41, 115 |
| Update | 1b | If the protocol is for an update of a previous systematic review, identify as such | ☐ | ☒ | N/A |
| **Registration** | 2 | If registered, provide the name of the registry (e.g., PROSPERO) and registration number in the Abstract | ☒ | ☐ | 46, 136 |
| **Authors** | | | | | |
| Contact | 3a | Provide name, institutional affiliation, and e-mail address of all protocol authors; provide physical mailing address of corresponding author | ☒ | ☐ | 4-24 |
| Contributions | 3b | Describe contributions of protocol authors and identify the guarantor of the review | ☒ | ☐ | 265 |
| **Amendments** | 4 | If the protocol represents an amendment of a previously completed or published protocol, identify as such and list changes; otherwise, state plan for documenting important protocol amendments | ☐ | ☒ | N/A |
| **Support** | | | | | |
| Sources | 5a | Indicate sources of financial or other support for the review | ☒ | ☐ | 27 |
| Sponsor | 5b | Provide name for the review funder and/or sponsor | ☒ | ☐ | 27 |
| Role of sponsor/funder | 5c | Describe roles of funder(s), sponsor(s), and/or institution(s), if any, in developing the protocol | ☐ | ☒ | N/A |
| **INTRODUCTION** | | | | | |
| **Rationale** | 6 | Describe the rationale for the review in the context of what is already known | ☒ | ☐ | 79-115 |

BioMed Central The Open Access Publisher

For peer review only - http://bmjopen.bmj.com/site/about/guidelines.xhtml

| Section/topic | # | Checklist item | Information reported | | Line number(s) |
|---|---|---|---|---|---|
| | | | Yes | No | |
| **Objectives** | 7 | Provide an explicit statement of the question(s) the review will address with reference to participants, interventions, comparators, and outcomes (PICO) | ☒ | ☐ | 119, 151 |
| **METHODS** | | | | | |
| **Eligibility criteria** | 8 | Specify the study characteristics (e.g., PICO, study design, setting, time frame) and report characteristics (e.g., years considered, language, publication status) to be used as criteria for eligibility for the review | ☒ | ☐ | 147-175 |
| **Information sources** | 9 | Describe all intended information sources (e.g., electronic databases, contact with study authors, trial registers, or other grey literature sources) with planned dates of coverage | ☒ | ☐ | 140-145 |
| **Search strategy** | 10 | Present draft of search strategy to be used for at least one electronic database, including planned limits, such that it could be repeated | ☒ | ☐ | Appendix |
| ***STUDY RECORDS*** | | | | | |
| Data management | 11a | Describe the mechanism(s) that will be used to manage records and data throughout the review | ☒ | ☐ | 177-181 |
| Selection process | 11b | State the process that will be used for selecting studies (e.g., two independent reviewers) through each phase of the review (i.e., screening, eligibility, and inclusion in meta-analysis) | ☒ | ☐ | 182-187 |
| Data collection process | 11c | Describe planned method of extracting data from reports (e.g., piloting forms, done independently, in duplicate), any processes for obtaining and confirming data from investigators | ☒ | ☐ | 188-193 |
| **Data items** | 12 | List and define all variables for which data will be sought (e.g., PICO items, funding sources), any pre-planned data assumptions and simplifications | ☒ | ☐ | 194-213 |
| **Outcomes and prioritization** | 13 | List and define all outcomes for which data will be sought, including prioritization of main and additional outcomes, with rationale | ☒ | ☐ | 206-213 |
| **Risk of bias in individual studies** | 14 | Describe anticipated methods for assessing risk of bias of individual studies, including whether this will be done at the outcome or study level, or both; state how this information will be used in data synthesis | ☒ | ☐ | 214-224 |
| ***DATA*** | | | | | |
| **Synthesis** | 15a | Describe criteria under which study data will be quantitatively synthesized | ☒ | ☐ | 226-242 |
| | 15b | If data are appropriate for quantitative synthesis, describe planned summary measures, methods of handling data, and methods of combining data from studies, including any planned exploration of consistency (e.g., $I^2$, Kendall's tau) | ☒ | ☐ | 244 |
| | 15c | Describe any proposed additional analyses (e.g., sensitivity or subgroup analyses, meta- | ☒ | ☐ | 233 |

BioMed Central
The Open Access Publisher

| Section/topic | # | Checklist item | Information reported Yes | Information reported No | Line number(s) |
|---|---|---|---|---|---|
| | | regression) | | | |
| | 15d | If quantitative synthesis is not appropriate, describe the type of summary planned | ☒ | ☐ | 229 |
| **Meta-bias(es)** | 16 | Specify any planned assessment of meta-bias(es) (e.g., publication bias across studies, selective reporting within studies) | ☒ | ☐ | 233 |
| **Confidence in cumulative evidence** | 17 | Describe how the strength of the body of evidence will be assessed (e.g., GRADE) | ☒ | ☐ | 223 |

For peer review only



BioMed Central The Open Access Publisher
